# Early reverse remodeling of left heart morphology and function evaluated by cardiac magnetic resonance in hypertrophic obstructive cardiomyopathy after transapical beating-heart septal myectomy

**DOI:** 10.1186/s12968-023-00987-0

**Published:** 2023-11-27

**Authors:** Yun Zhao, Chenhe Li, Dazhong Tang, Yi Luo, Chunlin Xiang, Lu Huang, Xiaoyue Zhou, Jing Fang, Xiang Wei, Liming Xia

**Affiliations:** 1 Department of Radiology, Tongji Hospital, Tongji Medical College, Huazhong University of Science and Technology, Wuhan, China; 2 Department of Cardiovascular Surgery, Tongji Hospital, Tongji Medical College, Huazhong University of Science and Technology, Wuhan, China; 3 MR Collaboration, Siemens Healthineers Ltd., Shanghai, China

**Keywords:** Hypertrophic obstructive cardiomyopathy, cardiovascular magnetic resonance, Reverse remodeling, Transapical beating-heart septal myectomy

## Abstract

**Purpose:**

This study aimed to evaluate the early morphology and function of the left heart in hypertrophic obstructive cardiomyopathy (HOCM) after transapical beating-heart septal myectomy (TA-BSM) using cardiovascular magnetic resonance (CMR).

**Materials and methods:**

Between April 2022 and January 2023, HOCM patients who underwent CMR before and 3 months after TA-BSM were prospectively and consecutively enrolled in the study. Preoperative and postoperative cardiac morphological and functional parameters, including those for the left atrium (LA) and left ventricle (LV), were compared. The left ventricular remodeling index (LVRI) was defined as the ratio between left ventricular mass (LVM) and left ventricular end-diastolic volume (LVEDV). Healthy participants with a similar age and sex distribution were enrolled for comparison. Pearson or Spearman correlation analysis was used to investigate the relationships between the parameters and LVRI. Last, univariate and multivariate linear regression identified variables associated with the LVM index (LVMI) and LVRI.

**Results:**

Forty-one patients (mean age ± standard deviation, 46 ± 2 years; 27 males) and 41 healthy control participants were evaluated. Eighteen (44%) HOCM patients were classified as having a sigmoid septum, and 23 patients had a reverse septal curvature. LA volume, diameter and function were significantly improved postoperatively, but still worse than healthy controls (all p < 0.001). Compared to before the operation, left ventricular wall thickness, left ventricular ejection fraction (LVEF), LVMI, and LVRI decreased after TA-BSM (all p < 0.001). The left ventricular end-diastolic volume index (LVEDVI) and left ventricular end-diastolic diameter (LVEDD) decreased in patients with a sigmoid septum. However, LVEDVI and LVEDD increased in those with a reverse septal curvature (both p < 0.001). In addition, both preoperative and postoperative LVRI was positively correlated with LVMI (r = 0.734 and 0.853, both p < 0.001) and maximum wall thickness (r = 0.679 and 0.676, both p < 0.001), respectively. In the multivariable analysis, the weight of the resected myocardium (adjusted β = 0.476, p = 0.005) and △mitral regurgitation degree (adjusted β = − 0.245, p = 0.040) were associated with △LVRI. Last, the △LVOTG (adjusted β = 0.436, p = 0.018) and baseline LVMI (adjusted β = 0.323, p = 0.040) were independently associated with greater left ventricular mass regression after TA-BSM.

**Conclusion:**

CMR confirmed early reverse remodeling of left heart morphology and function in HOCM patients following TA-BSM.

## Introduction

Hypertrophic cardiomyopathy (HCM) is an autosomal dominant cardiomyopathy with an incidence of 0.2–0.5% and is characterized by asymmetric myocardial hypertrophy [1]. Left ventricular outflow tract (LVOT) obstruction is present in 75% of HCM patients [2]. A form of HCM known as hypertrophic obstructive cardiomyopathy (HOCM) often causes severe symptoms and decreased labor endurance, placing patients at high risk of sudden cardiac death (SCD) and progressive heart failure (HF) [3]. In addition, ventricular septal thickening leads to LVOT stenosis and an increased systolic pressure gradient, causing systolic anterior motion (SAM) and exacerbating LVOT obstruction. Excessive afterload leads to increased cardiomyocyte hypertrophy, increased fibrosis, reduced exercise, and, ultimately, reduced LVEF and HF.

The most effective treatment to relieve LVOT obstruction in HOCM and significantly reduce the risk of SCD is surgery [4, 5]. Transapical beating-heart septal myectomy (TA-BSM) [6] is a new type of precision ventricular septal myectomy characterized by being minimally invasive and independent of the need for extracorporeal circulation. The procedure was enabled by an innovative beating-heart myectomy device (BMD). Mini-thoracotomy was performed in the fifth or sixth intercostal space at the left midclavicular line, identifying the apical position by echocardiography. Subsequently, the BMD was positioned in the LVOT after apical puncture. BMD is immobilized on the hypertrophic myocardium with negative pressure suction to allow for precise resection under Doppler ultrasound guidance.

Surgery relieves the mechanical stress overload of the left ventricle (LV) and greatly improves the LV hyperdynamic state. It is essential to evaluate left heart morphology and function accurately after surgery. Cardiovascular magnetic resonance imaging (CMR) has been widely used for the non-invasive evaluation of cardiac morphology and function, particularly considering that it is the gold standard for cardiac function. Previous studies have shown that transthoracic myectomy effectively improves patients' clinical symptoms and cardiac function [7–9]. However, few studies have evaluated this new, minimally invasive procedure. This study aimed to evaluate the morphological and functional parameters of the left heart before and after TA-BSM in HOCM patients using CMR and to further explore the factors associated with reverse remodeling.

## Materials and methods

### Study cohort

**Fig. 1:**
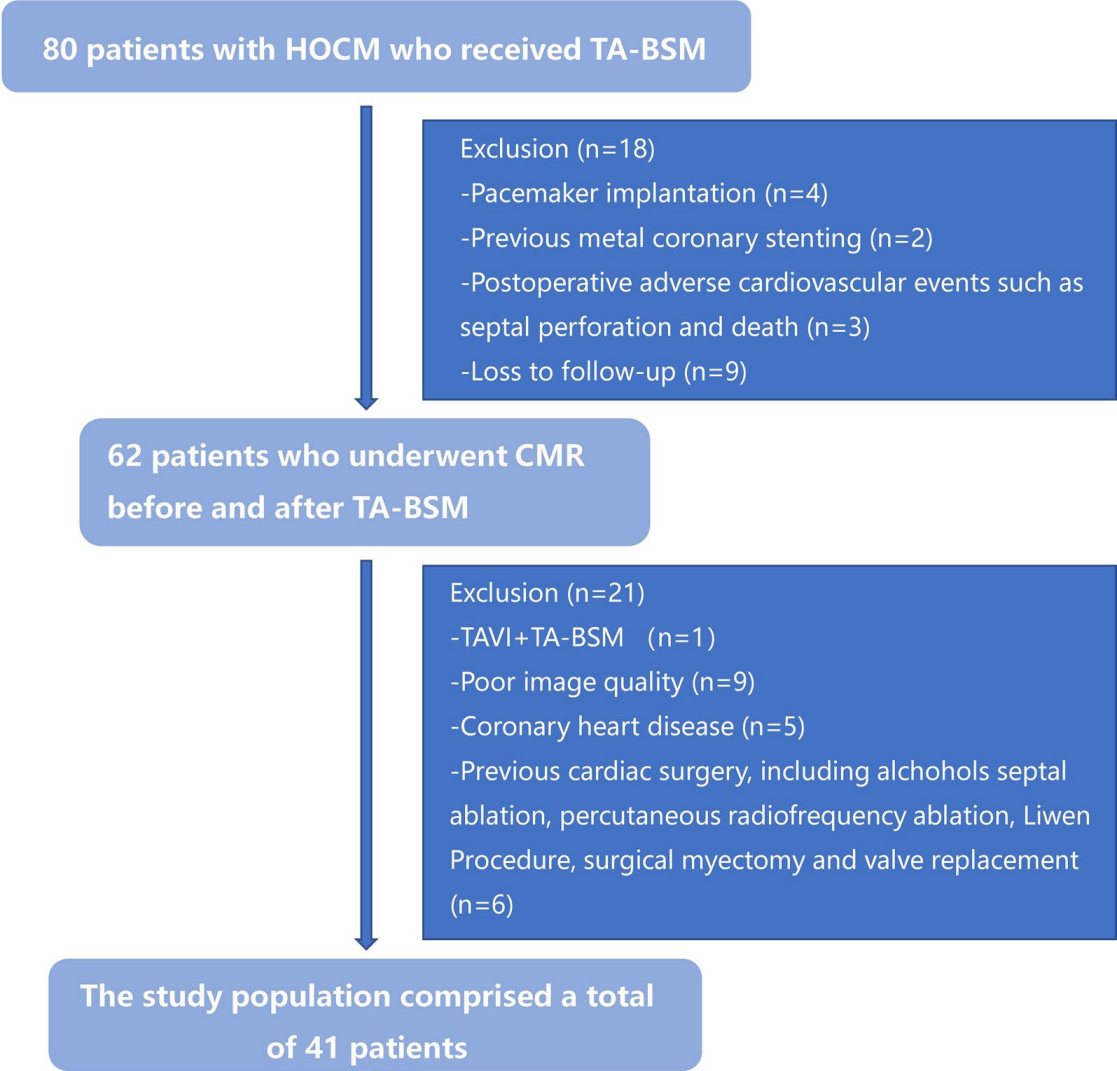
Flowchart showing patient inclusion in the study. *HOCM* hypertrophic obstructive cardiomyopathy, *TA-BSM* transapical beating-heart septal myectomy, *CMR* cardiovascular magnetic resonance, *TAVI* transcatheter aortic valve implantation

We experimentally performed 10 cases of TA-BSM approved by the Tongji Hospital ethics committee and collected clinical data from the initial 10 HOCM patients as a pre-test result. The sample size was calculated according to the formula of the paired experimental design, taking into account the actual clinical surgical volume of this procedure ($n=\frac{{\left( {Z}_{\frac{\alpha }{2}}+{Z}_{\beta }\right)}^{2}{* \sigma }^{2}}{{\delta }^{2}}$, α = 0.05, β = 0.1). Finally, the study prospectively enrolled 80 HOCM patients who underwent TA-BSM in Tongji Hospital from April 2022 to January 2023 (Fig. 1). All patients were diagnosed as having HCM based on the published guidelines (maximum wall thickness ≥ 15 mm or ≥ 13 mm in patients with a family history of HCM, in the absence of other cardiovascular diseases) [1]. The indications for TA-BSM were: (1) resting or provoked left ventricular outflow tract pressure gradient (LVOTG) ≥ 50 mmHg, and (2) severe symptoms or poor response to medical therapy. The major exclusion criteria were: (1) failure to undergo complete CMR examinations before and three months after TA-BSM (e.g., a previous pacemaker or metal stent implantation, postoperative adverse events, and missed appointments), (2) poor image quality due to arrhythmias, (3) other procedures performed simultaneously, such as transcatheter aortic valve implantation, (4) combined severe coronary artery disease [coronary artery stenosis ≥ 50%], (5) previous cardiac surgery, including alcohol septal ablation, percutaneous radiofrequency ablation, the Liwen procedure, surgical myectomy, and valve replacement. Patients with previous ventricular septal myectomy or valve repair or replacement were excluded. This prospective single-center study was approved by the Tongji Hospital ethics committee (approval numbers: 2022-S013, 2022-S013-1, 2022-S013-2, 2022-S013-3, and 2022-S013-4). Informed consent was obtained from all patients for this study.

### Echocardiography

Standard transthoracic echocardiography (TTE) was performed to evaluate LVOT stenosis within one week before TA-BSM and to assess the surgical outcome three months after the operation. Conventional cardiac parameters were measured by two experienced echocardiographers. The peak LVOT velocity was acquired by continuous-wave Doppler echocardiography in the apical long-axis or five-chamber view in the resting and provoked states, respectively. The LVOT peak gradient was calculated using the simplified Bernoulli equation. Assessments of LV diastolic function performed were the ratio between early mitral inflow velocity and early diastolic mitral annular velocity (E/e') and mitral inflow early-to-late filling velocity ratio (E/A). Mitral regurgitation was graded as 0 (none), 1 + (mild), 2 + (moderate), 3 + (moderate to severe), 4 + (severe) [10]. Systolic anterior motion (SAM) was graded as 0 (none), 1 (leaflet-septal distance > 10 mm), 2 (leaflet–septal distance ≤ 10 mm but no leaflet-septal contact), 3 (leaflet-septal contact < 30% of the systolic duration), 4 (leaflet-septal contact ≥ 30% of the systolic duration), according to the recommendations of the American Society of Echocardiography [11].

### Magnetic resonance imaging

All patients underwent a standard CMR examination on a 3T system (MAGNETOM Skyra, Siemens Healthcare, Erlangen, Germany). Cine images (four-chamber, three-chamber, and short-axial views) were acquired with ECG-gated and breath-holding using a segmented, balanced, steady-state free-precession sequence. Short-axial cine covered the LV from the apex to the mitral valval ring. The typical parameters were: section thickness = 8 mm, section gap = 2 mm, echo time = 1.39 ms, repetition time = 3.2 ms, field of view = 360 × 360 mm^2^, matrix size = 189 × 154, flip angle = 46°, temporal resolution 32 ms, and calculated phases 25.

### Data analysis: function and morphology

All CMR function analyses were performed using commercial cardiac software (CVI 42, version 5.14.0, Circle Cardiovascular Imaging Inc., Calgary, Alberta, Canada). Epicardial and endocardial LV contours from the base to the apex were automatically delineated on short-axial cines. The maximum wall thickness of surgical and non-surgical segments was measured manually according to the sixteen American Heart Association (AHA) segments. Left ventricular end-diastolic diameter (LVEDD) was measured from anteroseptal wall to inferolateral wall in the middle short-axial slice at the end-diastolic phase. Left ventricular end-diastolic volume (LVEDV), left ventricular end-systolic volume (LVESV), stroke volume (SV), cardiac output (CO), left ventricular ejection fraction (LVEF), and left ventricular mass (LVM) were obtained through end-diastolic and end-diastolic delineation. In addition, LVEDV, LVESV, CO, and LVM were indexed to the body surface area. The left ventricular remodeling index (LVRI) was calculated as the ratio between LVM and LVEDV (LVRI = LVM/LVEDV) [12, 13] which reflects the interplay between morphology and function during cardiac remodeling.

The left atrial (LA) anteroposterior and LA left–right diameters were obtained on four- and three-chamber cine images, respectively. The maximum LA volume (LAV_max_) and minimum LA volume (LAV_min_) were acquired with a combination of two- and four-chamber cine images at the end of ventricular systole and diastole. The left atrial ejection fraction (LAEF) was calculated as follows:

$$LAEF=\frac{\mathrm{LAV_{max} }-\mathrm{LAV_{min}}}{\mathrm{LAV_{max}}} \times 100\%,$$

### Statistical analysis

Statistical analysis of preoperative and postoperative CMR data was performed using SPSS statistical software (version 25.0, IBM SPSS Inc., Chicago, IL, USA). Continuous and normally distributed data were expressed as means ± standard deviations (SD), while medians [interquartile range (Q1, Q3)] were used for variables with non-normal distributions. Categorical variables were presented as percentage frequencies and compared using the chi-squared test. Parameters at baseline and follow-up CMR were compared using the paired t-test if the differences conformed to a normal distribution. Otherwise, non-normally distributed variables were compared using paired Wilcoxon signed-ranks test. In addition, comparisons of normally distributed continuous variables between HOCM patients and the general population were performed using the independent sample t-test. Mann–Whitney U test were used for comparation of non-normally distributed continuous variables. Statistical significance was indicated by p < 0.05.

The change in each variable was denoted by △, equal to the preoperative value minus the postoperative value. The Pearson correlation coefficient (r) and Spearman correlation coefficients (r_s_) were used to investigate the relationships between the parameters. Predictors of the left ventricular mass index (LVMI) reduction and LVRI were calculated using a stepwise multiple linear regression model entered as covariate factors. Last, variables with p < 0.05 were entered into the multivariable analysis.

Twenty patients were random selected from HOCM patients and controls respectively for the intra- and interobserver reproducibility assessment of CMR parameters by the intraclass correlation coefficient (ICC). All measurements were performed by two observers who were unaware of the values obtained during the selection process. The intra-observer measurements were repeated after 2 weeks by observers unawarded of the previous data.

## Results

### Patient characteristics

Forty-one HOCM patients were enrolled in this study, with a mean age of 45.9 ± 2.4 years. A total of 65.9% were male. The baseline characteristics of the 41 HOCM patients are listed in Table 1. There were 18 patients with a sigmoid septum and 23 with a reverse septal curvature. The primary symptom was dyspnea (80.5%), and the incidence of syncope was 22.0%. Preoperative New York Heart Association (NYHA) functional class 3 and above was present in 58.6% of patients.

**Table 1:** Demographic characteristics and clinical parameters associated with TA-BSM

Parameters	HOCM (n = 41)	Controls (n = 41)	p-value
Age (y)	46 ± 2.4	45 ± 2.4	0.81
Male (n (%))	27 (66)	29 (71)	0.64
BSA (m^2^)	1.89 ± 0.04	1.73 ± 0.03	0.08
Family history of HCM (n (%))	8 (19.5)	–	–
Medication use (n (%))			
β-blockers	32 (78)	–	–
Diltiazem	22 (54)	–	–
Chest pain (n (%))	24 (59)	–	–
Dyspnea (n (%))	33 (81)	–	–
Syncope (n (%))	9 (22)	–	–
Amaurosis (n (%))	15 (37)	–	–
Palpitations (n (%))	26 (63)	–	–
Postprandial symptom aggravation (n (%))	33 (81)	–	–
NYHA class (n (%))			
I	0 (0)	–	–
II	17 (42)	–	–
III	22 (54)	–	–
IV	2 (4.9)	–	–
Kansas City Cardiomyopathy Questionnaire Score	63 ± 2.5	–	–
6-min walking distance (m)	327 ± 15.8	–	–
Serum markers of myocardial injury			
NT-proBNP (pg/mL)	1161 (481, 3862)	–	–
cTnI (ng/ml)	48.6 (14.2, 402)	–	–
CK-MB (ng/ml)	2.2 (1.4, 3.7)	–	–
Electrocardiogram			
ST-T abnormality (n (%))	36 (88)	–	–
Rv5 + Sv1 (mV)	5.1 ± 0.3	–	–

*BSA* body surface area, *HCM* hypertrophic cardiomyopathy, *NYHA* New York Heart Association, *CK-MB* creatine kinase-myocardial band, *NT-proBNP* N-terminal pro-hormone brain natriuretic peptide, *cTnI* cardiac troponin I

### Cardiac surgery

**Fig. 2:**
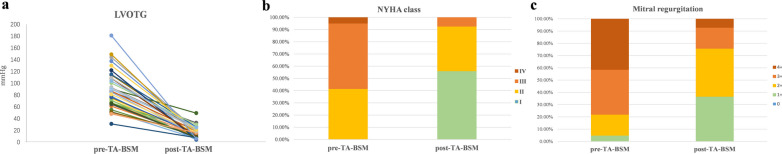
Comparison of LVOTG (**a**), NYHA class (**b**), and mitral regurgitation (**c**). LVOTG, outflow tract pressure gradient; NYHA, New York Heart Association

All patients underwent TA-BSM performed by the same cardiac surgeon with 25 years of experience. Minimally invasive myocardial resection without extracorporeal circulation was performed to remove abnormal hypertrophic myocardium. The mean myocardial resection mass was 5.6 (3.3, 10.6) g. Almost all patients experienced an improvement in symptoms after TA-BSM, and intraoperative catheter manometry showed a decrease in LVOTG to normal levels (< 30 mmHg). A total of 92.7% of postoperative patients were classified as NYHA I or II compared to 41.5% before myectomy (Fig. 2b).

### Echocardiography

The echocardiographic results are summarized in Table 2. The TTE showed that the LVOTG of all HOCM patients was significantly reduced after TA-BSM (89 ± 4.9 mmHg vs. 16 ± 1.4 mmHg, p < 0.001) (Fig. 2a). Mitral regurgitation occurred in all patients, of which moderate to severe accounted for 78.0% preoperatively and decreased to 7.3% postoperatively. (Fig. 2c). Almost all patients presented with mitral systolic anterior motion (SAM). SAM grades 3–4 occurred in 80.7% of patients but decreased to 2.4% postoperatively.

**Table 2:** Echocardiographic parameters in HOCM group before and after TA-BSM

Parameters	Preoperative	Postoperative	p-value
LVOTG (mmHg)	89 ± 4.9	16 ± 1.4	< 0.001
SAM (n (%))			
0	0 (0)	3 (7.3)	–
1	2 (5)	33 (81)	–
2	10 (24)	4 (9.8)	–
3	9 (22)	1 (2.4)	–
4	20 (49)	0 (0)	–
Mitral regurgitation (n (%))			
0	0 (0)	15 (37)	–
1 +	2 (4)	16 (39)	–
2 +	7 (17)	7 (17)	–
3 +	15 (37)	3 (7.3)	–
4 +	17 (42)	0 (0)	–
E/A (n (%))	1.1 (0.9,1.6)	0.9 ± 0.05	0.001
E/e′ (n (%))	17 (13, 23)	15 ± 0.7	0.002

*LVOTG* left ventricular outflow tract pressure gradient, *SAM* systolic anterior motion, *LVOT* left ventricular outflow tract

### Changes in LA morphology and function

As shown in Table 3, LA anteroposterior diameters, LA left–right diameters, LAV_max_, and LAV_min_ were significantly decreased postoperatively (all p < 0.001). LAEF increased postoperatively and was slightly lower than that of the healthy controls [pre-TA-BSM, 48 ± 1.4% vs. post-TA-BSM, 54 ± 1.5% (p < 0.001), Controls, 63 ± 1.2% (both p < 0.001 for pre-TA-BSM and post-TA-BSM)].

**Table 3:** Left atrial parameters in HOCM and control groups before and after TA-BSM

Parameters	Preoperative	Postoperative	Controls	p-value for pre- and post- operative
LA anteroposterior diameter (mm)	43 ± 1.2	37 ± 1.1	31 ± 0.6	< 0.001
LA left–right diameter (mm)	50 ± 1.2	43 ± 0.9	39 ± 0.7	< 0.001
LAVmax (ml)	108 ± 6.8	74 ± 4.7	53 ± 2.1	< 0.001
LAVmin (ml)	61 ± 4.7	39 ± 2.9	21 ± 1.2	< 0.001
LAEF (%)	48 ± 1.4	54 ± 1.5	63 ± 1.2	< 0.001

*LA* left atrium, *LAV* left atrial volume, *LAEF* left atrial ejection fraction

### Changes in LV morphology and function

**Fig. 3:**
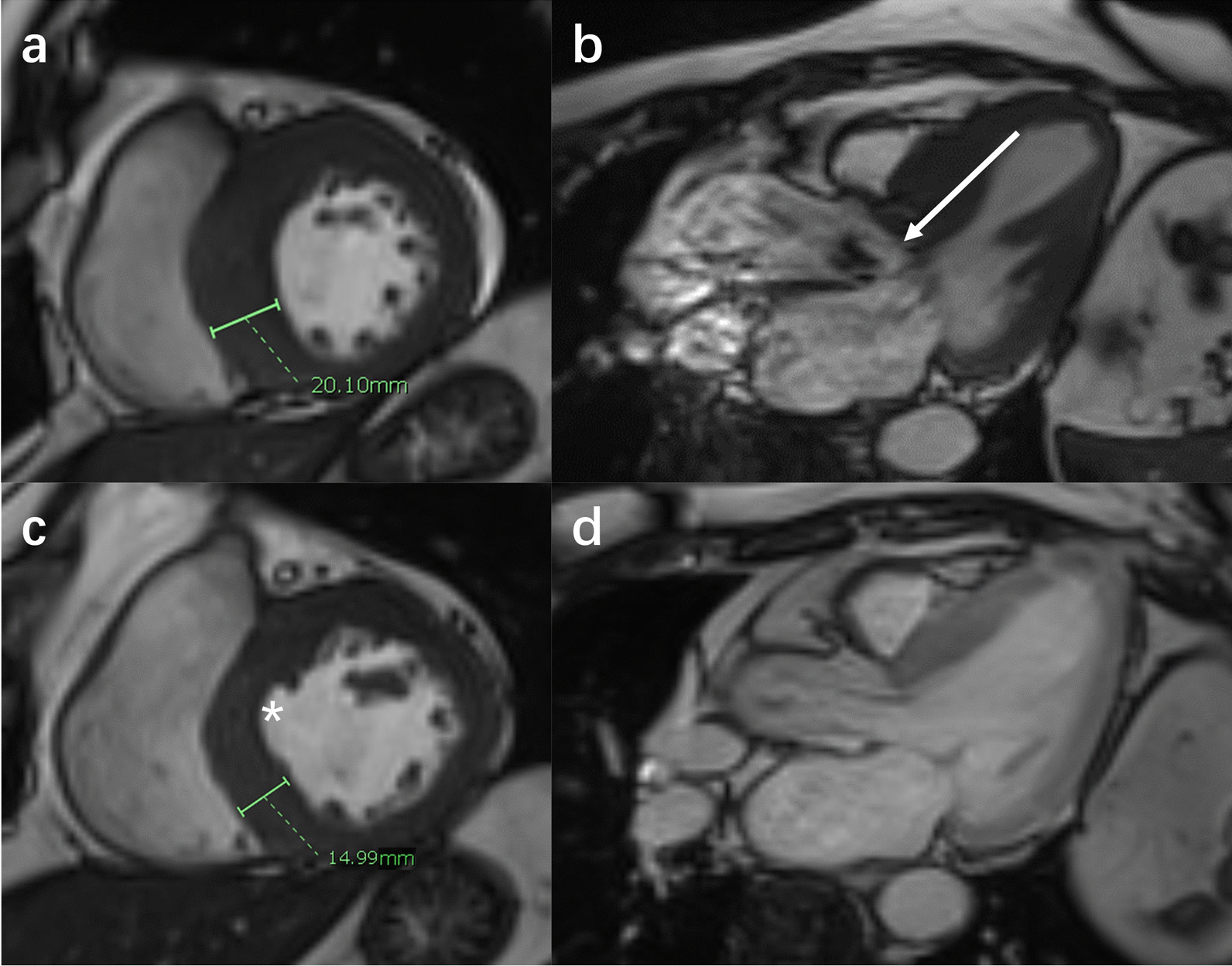
The SA cines (**a**, **c**) and three-chamber (**b**, **d**) images of a 53-year-old woman with HOCM. High-speed blood flow (white arrow) can be seen in the narrow left ventricular outflow tract preoperatively. The obstruction is relieved after TA-BSM. Part of the basal anterior ventricular septum myocardium (white star) was surgically removed. The basal posterior ventricular septum was not surgically removed, and its thickness was reduced from (**a**) 20.1 mm to (**c**) 14.9 mm three months after TA-BSM. SA, short-axis; HOCM, hypertrophic obstructive cardiomyopathy; TA-BSM, transapical beating-heart septal myectomy

The LV morphological and functional parameters of the study groups are displayed in Table 4. SV, CI, LVMI, LVRI, LVEF, maximum wall thickness, maximum wall thickness in non-surgical segments, and LVMI decreased after TA-BSM (all p < 0.05). However, SV, LVMI, LVRI, maximum wall thickness, and non-surgical segment maximum wall thickness were still higher than in the normal controls (all p < 0.05). A representative case of cine images before and after TA-BSM is shown in Fig. 3. After the procedure, the thickness of the myocardial segment without surgical resection decreased significantly [pre-TA-BSM, 22 ± 1.0 mm vs. post-TA-BSM, 16 ± 0.7 mm (p < 0.001), Controls, 9.1 ± 0.1 mm (both p < 0.001)], and the high-speed blood flow signals due to LVOT obstruction disappeared.

**Table 4:** Left ventricular parameters in HOCM and control groups before and after TA-BSM

Parameters	Preoperative	Postoperative	Controls	p-value for pre- and postoperative
LVEDVI (ml/m^2^)	82 ± 3.1	82 ± 2.3	73 ± 1.8	0.85
LVESVI (ml/m^2^)	28 ± 2.2	33 ± 1.5	29 ± 0.8	< 0.001
SV (ml)	102 ± 3.7	92 ± 3.5	78 ± 2.2	< 0.001
CI (ml/min/m^2^)	3.6 ± 0.1	3.2 ± 0.1	3.4 (2.7, 3.6)	0.002
LVEF (%)	67 ± 1.2	60 ± 1.0	61 ± 0.6	< 0.001
LVMI (g/m^2^)	94 (70, 119)	89 ± 4.5	44 ± 1.0	< 0.001
LVRI	1.24 ± 0.06	1.07 ± 0.04	0.600 ± 0.01	< 0.001
LVEDD	48 ± 1.1	50 ± 0.7	47 ± 0.5	0.089
Maximum wall thickness (mm)	23 ± 1.0	16 ± 0.7	9.1 ± 0.1	< 0.001
Maximum wall thickness in non-surgical segments (mm)	22 ± 1.0	16 ± 0.7	–	< 0.001
T1 value (ms)	1313 ± 7.5	1283 ± 7.6	1214 ± 3.6	< 0.001
T2 value (ms)	41.3 ± 0.4	40.3 ± 0.3	34.5 ± 0.1	0.025

*LVEDVI* left ventricular end-diastolic volume index, *LVESVi* left ventricular end-systolic volume index, *SV* stroke volume, *CI* cardiac index, *LVEF* left ventricular ejection fraction, *LVMI* left ventricular mass index, *LVRI* left ventricular remodeling index, *LVEDD* left ventricular end-diastolic diameter

**Fig. 4:**
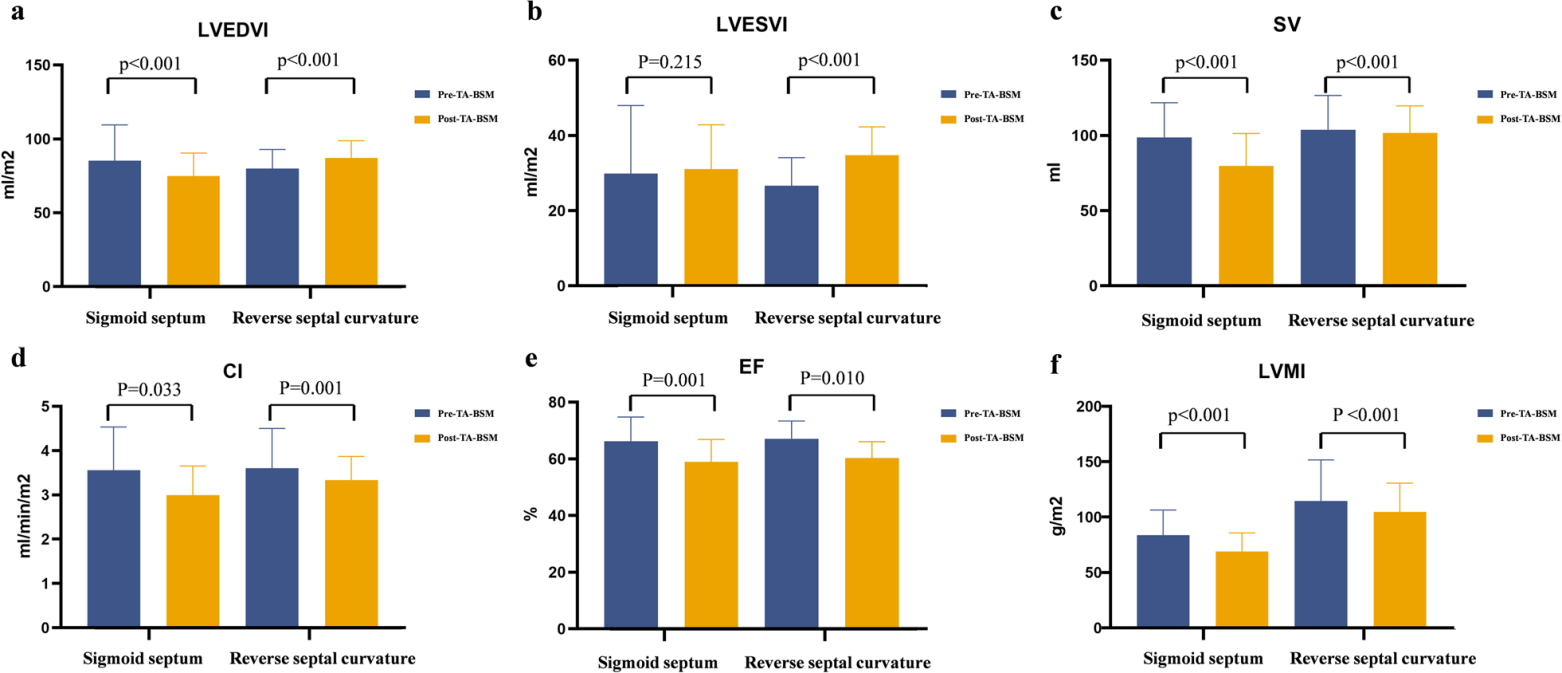
Preoperative and postoperative comparisons of LVEDVI (**a**), LVESVI (**b**), SV (**c**), CI (**d**), LVEF (**e**), and LVMI (**f**) between the two subgroups. *LVEDVI* left ventricular end-diastolic volume index, *LVESVI* left ventricular end-systolic volume index, *SV* stroke volume, *CI* cardiac index, *LVEF* left ventricular ejection fraction, *LVMI* left ventricular mass index

There was no significant preoperative difference between the two subgroups in the function parameters. Patients with a reverse septal curvature had a significantly higher LVMI and maximum wall thickness than patients with a sigmoid septum preoperatively (121 ± 6.5 g/m^2^ vs. 71 (68, 107) g/m^2^, 26 ± 1.1 mm vs. 19 ± 1.0 mm, both p < 0.001), but their LVEDD was smaller (45 ± 1.1 mm vs. 51 (48, 57) mm, p = 0.001). In addition, SV, CI, LVEF, LVMI, LVRI, maximum wall thickness, and maximum wall thickness in the non-surgical segments were significantly reduced postoperatively in both subgroups (all p < 0.05). However, the left ventricular end-diastolic volume index (LVEDVI) and LVEDD decreased in patients with a sigmoid septum postoperatively (pre-TA-BSM, 83 ml/m^2^ vs. post-TA-BSM, 73 ml/m^2^, pre-TA-BSM, 51 (48, 57) mm vs. post-TA-BSM, 47 (43, 50) mm, both p < 0.001) but increased in patients with reverse septal curvature (pre-TA-BSM, 80 ml/m^2^ vs. post-TA-BSM, 87 ml/m^2^, pre-TA-BSM, 45 ± 1.1 mm vs. post-TA-BSM, 51 ± 0.9, both p < 0.001) (Fig. 4 and Table 5).

**Table 5:** CMR parameters in sigmoid and reverse septal curvature subtypes of HOCM

Parameters	Sigmoid septum (n = 18)		Reverse septal curvature (n = 23)	
	Preoperative	Postoperative	Preoperative	Postoperative
LVEDVI (ml/m^2^)	83 (73, 89)	73 (62, 81)*	80 ± 2.7	87 ± 2.4*
LVESVI (ml/m^2^)	25 (23, 31)	29 (25, 32)	27 ± 1.6	35 ± 1.5*
SV (ml)	99 ± 5.4	80 ± 5.1*	104 ± 5.0	102 ± 3.7*
CI (ml/min/m^2^)	3.6 ± 0.2	2.9 ± 0.2*	3.6 ± 0.2	3.3 ± 0.1*
LVEF (%)	66 ± 2.0	59 ± 1.9*	67 ± 1.3	60 ± 1.1*
LVMI (g/m^2^)	71 (68, 107)	69 ± 3.9*	121 ± 6.5^#^	104 ± 5.4*
LVRI	1.00 ± 0.04	0.93 ± 0.04*	1.43 ± 0.08	1.19 ± 0.04*
LVEDD (mm)	51 (48, 57)	46 ± 2.6*	45 ± 1.1^#^	51 ± 0.8*
Maximum wall thickness (mm)	19 ± 1.0	13 ± 0.7*	26 ± 1.1^#^	19 ± 0.7*
Maximum wall thickness in non-surgical segments (mm)	18 ± 1.1	12 ± 0.8*	26 ± 1.0^#^	19 ± 0.7*

*LVEF* left ventricular ejection fraction, *LVMI* left ventricular mass index, *LVEDVI* left ventricular end-diastolic volume index, *LVESVI* left ventricular end-systolic volume index, *SV* stroke volume, *CI* cardiac index, *LVRI* left ventricular remodeling index, *LVEDD* left ventricular end-diastolic diameter

^*^p < 0.05 compared with pre-operation using paired-samples t-test or paired Wilcoxon signed-ranks test

^#^p < 0.05 compared with preoperative sigmoid septum group using independent samples t-test or Mann–Whitney U test

### Associations between conventional LVRI and LV parameters

**Fig. 5:**
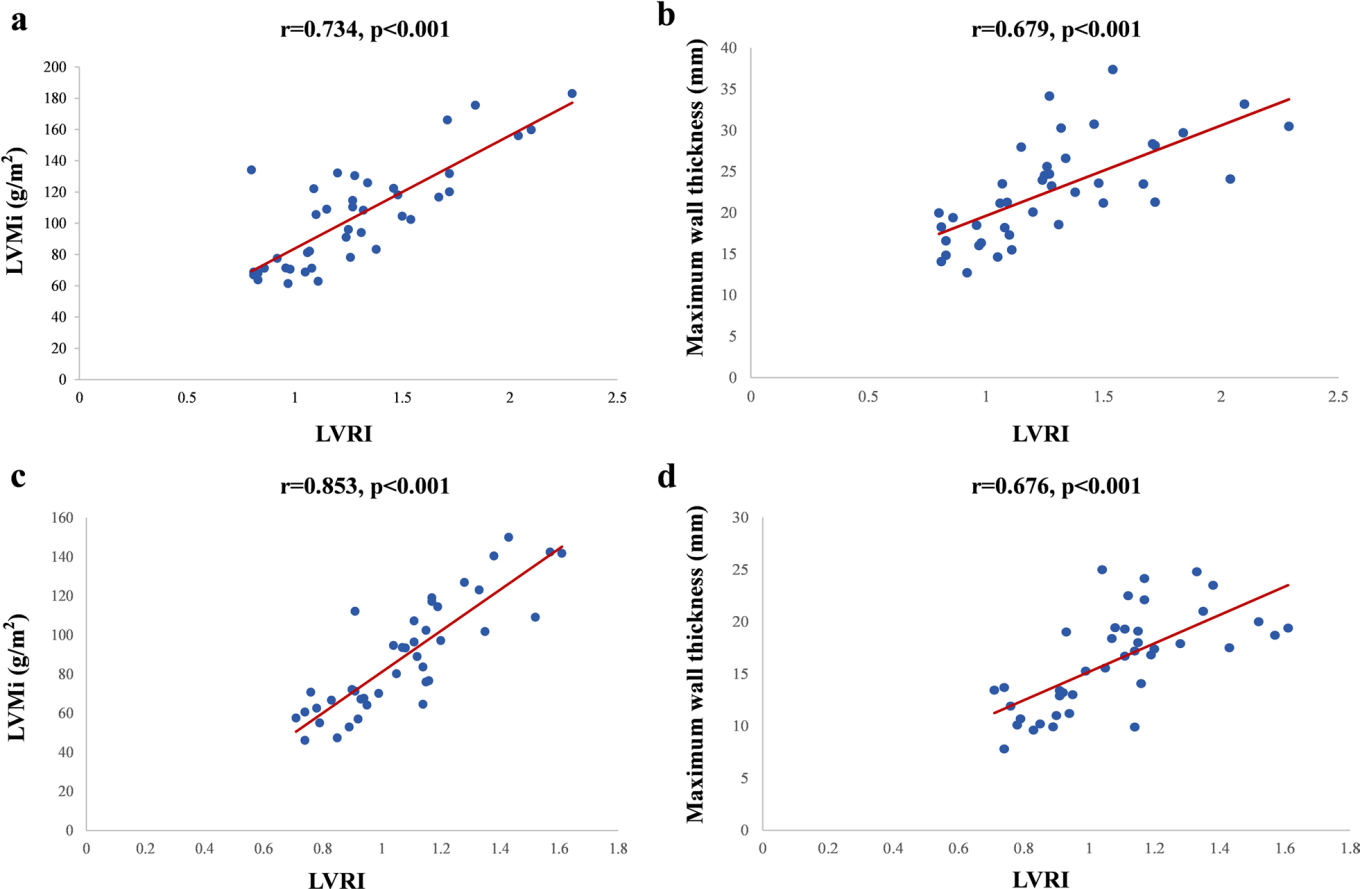
Scatterplots show significant preoperative and postoperative correlation between LVMI and the maximum wall thickness on LVRI, respectively, in all HOCM patients. *LVMI* left ventricular mass index, *LVRI* left ventricular remodeling index

The correlations between LVRI and conventional parameters are shown in Fig. 5. Among the morphological and functional parameters, LVMI and maximum wall thickness were significantly correlated with LVRI preoperatively (r_s_ = 0.734 and r = 0.679, both p < 0.001). After TA-BSM, both remained significantly associated with LVRI. The correlation between LVMI and LVRI was enhanced (r = 0.853, p < 0.001), but the correlation between maximal wall thickness and LVRI did not change significantly (r = 0.696, p < 0.001).

### Predictors of reverse remodeling

Clinical variables, including patient demographics, echocardiographic parameters, and CMR measurement parameters, were analyzed to identify the predictors of reverse remodeling. In the univariate analysis, the weight of the resected myocardium, maximum wall thickness, △SAM, △LVOTG, baseline LVEDVI, and baseline LVMI appeared to predict a reduction in LVMI (all p < 0.05). However, in the multivariate analysis, only △LVOTG (adjusted β = 0.323, p = 0.018) and baseline LVMI (adjusted β = 0.436, p < 0.040) were independent predictors of LV mass regression (Table 6). In addition, the weight of the resected myocardium (adjusted β = 0.476, p = 0.005) and △mitral regurgitation degree (adjusted β = − 0.245, p = 0.040) were associated with △LVRI.

**Table 6:** Predictors of reverse remodeling of LVMI reduction and LVRI after TA-BSM by multivariate analysis

Variables	LVMI reduction				LVRI			
	Univariate		Multivariate		Univariate		Multivariate	
	r	p	Adjusted β	p	r	p	Adjusted β	p
Age	0.049	0.759			− 0.003	0.173		
Male	0.019	0.907			− 0.004	0.949		
BSA	0.011	0.945			0.075	0.559		
Family history of HCM	0.132	0.832			− 0.046	0.569		
Preoperative β-blockers	− 0.030	0.853			0.199	0.007	0.194	0.110
Preoperative diltiazem	− 0.190	0.235			0.053	0.404		
ST-T abnormal	0.056	0.729			0.049	0.619		
Rv5 + Sv1	0.291	0.077			0.028	0.089		
6-min walking distance	0.085	0.602			< 0.001	0.711		
△NYHA class	0.045	0.781			0.026	0.520		
Weight of resected myocardium	0.477	0.002	0.107	0.521	0.019	< 0.001	0.476	0.005
Mitral E/A	− 0.062	0.700			− 0.073	0.253		
Maximum wall thickness	0.315	0.045	− 0.081	0.936	0.019	< 0.001	0.220	0.171
△Mitral regurgitation degree	0.217	0.173			− 0.069	0.013	− 0.245	0.040
△SAM	0.372	0.017	0.219	0.828	0.012	0.708		
△LVOTG	0.667	< 0.001	0.436	0.018	0.001	0.381		
Baseline LVEDVI	0.412	0.007	0.127	0.346	− 0.003	0.063		
Baseline LVESVi	0.257	0.105			− 0.002	0.160		
Baseline LVMI	0.615	< 0.001	0.323	0.040	0.002	0.020	− 0.104	0.496

*BSA* body surface area, *HCM* hypertrophic cardiomyopathy, *NYHA* New York Heart Association, *LVOTG* left ventricular outflow tract pressure gradient, *LVEDVI* left ventricular end-diastolic volume index, *LVESVi* left ventricular end-systolic volume index, *LVMI* left ventricular mass index

### Reproducibility of CMR parameters

The LA and LV parameters in the two subgroups before and after TA-BSM showed excellent reproducibility (intra-observer ICC: 0.90–0.99; inter-observer ICC: 0.92–0.99).

## Discussion

In this study, we performed a pre- and postoperative structural and functional analysis of the left heart during the TA-BSM procedure. Our study demonstrated the following three findings: (1) left heart morphology and function partially recovered after relieving the LVOT obstruction; (2) morphological remodeling after TA-BSM differs between the various types of hypertrophy; (3) baseline LVMI and maximum wall thickness were significantly correlated with LVRI both preoperatively and postoperatively; and (4) the weight of the resected myocardium and △mitral regurgitation degree might be associated with remodeling after TA-BSM. In addition, △LVOTG and baseline LVMI might be potential independent predictors of LV mass regression.

There are two primary mechanisms of myocardial hypertrophy. The first is when genetic factors lead to cardiomyocyte hypertrophy and extracellular matrix fibrosis; the other is secondary hypertrophy caused by increased afterload [14]. Genes are the initiating factors that drive cardiomyocyte hypertrophy and extracellular matrix proliferation. Hypertrophic myocardium has hypercontractility, resulting in increased power output [15]. However, the normal turbulent state of blood in the aorta is disturbed due to LVOT obstruction, particularly in patients with basal ventricular septal hypertrophy. Abnormal ejection and the increased contractile capacity of cardiomyocytes further increase left ventricular systolic pressure. Abnormal circulation in the heart chambers and outflow tract prompts further myocardial remodeling and aggravation of fibrosis.

Initially, myocardial remodeling was used to describe changes in ventricular expansion and cardiomyocytes after myocardial infarction [16]. Ventricular remodeling describes changes in the overall geometry and deterioration of ventricular contractile function [14]. The process of left ventricular remodeling is due to prolonged volume or pressure overload, myocardial cell degeneration, and changes in the extracellular composition [17]. Subsequently, the end-diastolic pressure–volume relationship in the ventricle was introduced to evaluate structural remodeling [18].

Previous clinical studies have shown that drug and cardiac resynchronization therapy can somewhat improve cardiac size and function [19]. Currently, reverse remodeling of the ventricle has gradually become the focus of attention in heart disease treatment. This process refers to the normalization of cardiomyocytes and ventricular geometry and is related to the positive changes in cardiomyocyte molecular metabolism and the extracellular matrix [19–21]. The changes, including reductions in LV volume and mass, restoration of more normal ventricular geometry, and improved LVEF, are consistently associated with reductions in morbidity and mortality [19].

The phenotypic variation in HOCM patients results from a combination of many factors, including afterload, wall stress, and microvascular dysfunction [22]. Finally, their corresponding pathological changes include tissue-level inflammation [23, 24]. Collagen deposition and cross-linking are early and essential manifestations of HCM associated with adverse cardiac remodeling and clinical outcomes [25, 26]. We found that myocardial thicknesses in the non-operative segments were also significantly reduced after the surgical relief of LVOT obstruction and consequent pressure gradient reduction. This process may be related to decreased intra- and extracellular inflammation in the myocardium following a mechanical load reduction. At the same time, we observed a mild decrease in both postoperative T1 and T2 values, suggesting reverse histological remodeling. Reversible myocardial edema and collagen deposition may be necessary for reverse remodeling of the myocardium.

Similar to our results, a study of 44 patients with obstructive HCM reported a significant degree of regional LV thickness regression in all segments [27]. Another study using cardiac computed tomography similarly found that all segments except the basal inferior and inferolateral regions showed significantly decreased wall thickness [28]. Together, these studies and ours show that proper septal myectomy might induce remodeling of the entire left ventricle, not just the resected area.

During short-term follow-up, we found that the left ventricular myocardial mass was much greater than that resected during TA-BSM, strongly confirming left ventricular reverse remodeling. Previous studies have shown that abnormal gene expression persists despite ventricular function and structure improvements after reduced mechanical stress [29, 30]. This finding explains the improvement in postoperative cardiac structure and function and that it does not fully return to normal levels. This phenomenon may be associated with incomplete induction of transcriptional recovery after mechanical unloading [31].

The primary pathophysiology in patients with sigmoid septum is LVOT obstruction. This process results in blood stagnation and increased pressure in the left ventricular chamber. The LV is progressively enlarged due to prolonged hyper-stress and is spherically dilated in most patients. In addition, patients in the reverse septal curvature group also experience diffuse hypertrophy of most segments, mainly manifesting as diastolic dysfunction. Systolic ventricular occlusion is common due to the space occupied by the over-thickened myocardium. Previously, there were fewer studies on HCM subtypes. Individualized treatment for different hypertrophic forms is critical.

We also found that preoperative LVMI and ΔLVOTG correlated with LVMI reduction. Left ventricular hypertrophy, myocardial fibrosis, and the HF development are well-recognized [32]. Left ventricular hypertrophy is an independent predictor of cardiac mortality [33–35], considered a marker of disease development severity, and is associated with adverse clinical outcomes such as SCD [35, 36]. In our study, baseline LVMI was more likely to be an independent predictor of left ventricular mass regression than maximal wall thickness, perhaps because LVMI better reflects the extent and severity of complete myocardial remodeling. A greater preoperative LVMI means more adverse myocardial remolding at the cellular and molecular levels.

A study of 71 patients with ventricular septal myectomy showed that sufficient relief of obstruction and lower resected thickness lead to more favorable remodeling [9]. In addition, one echocardiographic study reported significant changes in LVOT gradients (≥ 10 mmHg) as the only variable independently associated with LAVI reverse remodeling [37]. Maximum ventricular wall thickness, LA diameter, and LVOT obstruction are major risk factors for the 2014 HCM SCD Risk Score and 2020 ACC/AHA HCM adverse outcomes [1, 4]. Our study demonstrated a significant reduction of these parameters after TA-BSM, symbolizing a good prognosis. Moreover, the degree of reverse remodeling may be a benign indicator of the reduction in the risk of HF, new-onset atrial fibrillation, ventricular arrhythmia, and even SCD. In contrast to our results, an investigation reported that lower baseline LVMI was independently associated with greater LV mass regression after alcohol septal ablation [38]. The phenomenon may be due to differences in the surgical approach and rationale, although both are used to relieve the obstruction in HOCM. Our study findings suggested that patients with a greater LVOTG reduction might benefit more from TA-BSM in reverse cardiac remodeling. Considering the clinical benefit of hemodynamic improvement and the potential impact of left ventricular remodeling, elimination of LVOT obstruction and adequate myocardial resection are essential to TA-BSM in HOCM.

## Limitations

This study has several limitations. First, this was a single-center study with a small sample size because the clinical application time of this minimally invasive technique was brief. Therefore, the study results might not be generalizable. Second, we excluded patients who failed to undergo a complete CMR before and after surgery, possibly leading to selection bias. Third, we did not perform a predictive analysis because there were few clinically malignant events, and this study was only a short-term follow-up study. Last, we did not study the characteristics of late gadolinium enhancement in the myocardium because some patients did not complete perfusion or enhancement examinations for various reasons, such as mild renal impairment.

## Conclusions

Our study summarizes the CMR findings indicative of left heart remodeling after TA-BSM. Following partial ventricular septal resection, LVOT obstruction was entirely relieved. The degree of left ventricular myocardial hypertrophy was reduced, including in the non-surgical segments. In addition, the structure and function of LA and LV were recovered to some extent. Future studies are needed to verify the long-term structural and functional changes of the left ventricular myocardium in patients after TA-BSM.

## Data Availability

The data from the study are not publicly available due to patient privacy concern but are available on reasonable request from the corresponding author.
